# Comparison of Endovascular Embolization Plus Simultaneous Microsurgical Resection vs. Primary Microsurgical Resection for High-Grade Brain Arteriovenous Malformations

**DOI:** 10.3389/fneur.2021.756307

**Published:** 2021-12-24

**Authors:** Mingze Wang, Fa Lin, Hancheng Qiu, Yong Cao, Shuo Wang, Jizong Zhao

**Affiliations:** ^1^Department of Neurosurgery, Beijing Tiantan Hospital, Capital Medical University, Beijing, China; ^2^China National Clinical Research Center for Neurological Diseases, Beijing, China; ^3^Center of Stroke, Beijing Institute of Brain Disorder, Beijing, China; ^4^Beijing Key Laboratory of Translational Medicine for Cerebrovascular Disease, Beijing, China

**Keywords:** arteriovenous malformation, Spetzler-Martin Grade, endovascular embolization, microsurgical resection, one-staged hybrid operation

## Abstract

**Aim:** It remains a challenge in surgical treatments of brain arteriovenous malformations (AVMs) in Spetzler-Martin Grade (SMG) IV and V to achieve both optimal neurological outcomes and complete obliteration. The authors reported a series of patients with AVMs in SMG IV and V who underwent a surgical paradigm of endovascular embolization and simultaneous microsurgical resection based on the one-staged hybrid operation.

**Methods:** Participants in the multicenter prospective clinical trial (NCT 03774017) between January 2016 and December 2019 were enrolled. Patients who received endovascular embolization plus microsurgical resection (EE+MRS) and those who received intraoperative digital subtraction angiography plus microsurgical resection (iDSA+MRS) were divided into two groups. Information on clinical features, operative details, and clinical outcomes were extracted from the database. Deterioration of neurological deficits (DNDs) was defined as the primary outcome, which represented neurological outcomes. The time of microsurgical operation and blood loss were defined as the secondary outcomes representing microsurgical risks and difficulties. Outcomes and technical details were compared between groups.

**Results:** Thirty-eight cases (male: female = 23:15) were enrolled, with 24 cases in the EE+MRS group and 14 in the iDSA+MRS group. Five cases (13.2%) were in SMG V and 33 cases (86.8%) were in SMG IV. Fourteen cases (36.8%) underwent the paradigm of microsurgical resection plus intraoperative DSA. Twenty-four cases (63.2%, *n* = 24) underwent the paradigm of endovascular embolization plus simultaneous microsurgical resection. Degradations of SMG were achieved in 15 cases. Of the cases, two cases got the residual nidus detected via intraoperative DSA and resected. Deterioration of neurological deficits occurred in 23.7% of cases (*n* = 9) when discharged, and in 13.5, 13.5, 8.1% of cases at the follow-ups of 3, 6, and 12 months, respectively, without significant difference between groups (*P* > 0.05). Intracranial hemorrhagic complications were reported in three cases (7.9%) of the EE+MRS group only. The embolization did not significantly affect the surgical time and intraoperative blood loss. The subtotal embolization or the degradation of size by 2 points resulted in no DNDs.

**Conclusions:** The paradigms based on the one-staged hybrid operation were practical and effective in treating high-grade AVMs. Appropriate intraoperative embolization could help decrease operative risks and difficulties and improve neurological outcomes.

## Introduction

The Spetzler-Martin Grading (SMG) system is widely used to evaluate therapeutic risks of brain arteriovenous malformations (AVMs) (1), and sorts AVMs into two classes: the low-grades (Grades I to III) and the high-grades (Grade IV and V). High-grade AVMs usually have more complicated features on location, angioarchitecture, and hemodynamics, which increases its hemorrhagic risks (2–4). With an annual hemorrhagic risk ranging from 1.5 to 2.7%, conventional treatments have not shown any significant superiority compared with surgical interventions (5–11). Surgical treatments on high-grade AVMs are necessary, especially for patients with long life expectancies. However, with concern to neurological outcomes, the optimized procedure or paradigm is being explored.

Microsurgical resection achieves the highest obliteration rate among treatments of AVMs. However, it sometimes fails to meet the requirement of preventing neurological deficits (12, 13). The incidence of neurological deficits is reported to be 31 and 37% in lesions of SMG IV and V, respectively (14). Binary and trinary multimodality therapeutic paradigms are proposed for the high-grades, consisting of microsurgical resection, endovascular intervention, and stereotactic radiosurgery (SRS). The multimodality paradigm, that consisted of endovascular embolization and subsequent stereotactic radiosurgery, could only achieve an obliteration rate ranging from 38 to 42% (15–18), and up to 44% by modifying SRS strategies (19). Another multimodality paradigm consisted of volume-staged SRS and subsequent microsurgical resection was reported to achieve an obliteration rate of 93.8% (20). The binary combination of endovascular embolization and microsurgical resection is the most widely used paradigm for treating high-grade AVMs (21, 22). The preoperative embolization could decrease the blood volume and velocity of the nidus, and reduce the risk and difficulty of the subsequent microsurgery. The paradigm results in a high obliteration rate with an acceptable incidence of neurological deficits. However, complications of endovascular intervention remain a shortcoming of solely performed endovascular embolization, such as catheter stinking, intraoperative hemorrhage, and embolization-induced normal perfusion pressure breakthrough (23). In addition, all of the staged paradigms have to face the risks of adverse events in the latency period, especially the hemorrhagic risk, which ranges from 1.1 to 3.3% per year (16–20), and remains unchanged, as long as AVMs have not been completely obliterated.

Since the timing of microsurgical resection after preoperative embolization remains unclear (24), the paradigm of endovascular embolization plus simultaneous microsurgical resection (AKA one-staged hybrid operation) could be a practical solution without the risks of solely performed embolization and the latency period. The objective of this study is to introduce the experience of applying this paradigm to treat high-grade AVMs in one-staged hybrid operations, and its technical details which could potentially improve the functional outcome of the high-grades.

## Methods

Data of patients with high-grade AVMs were extracted from the database of a multicenter prospective clinical trial (NCT 03774017) from January 2016 to December 2019 (24). Inclusion criteria included: 1) harboring AVMs of SMG IV or V; and 2) received endovascular interventions, including intraoperative digital subtraction angiography (iDSA) or embolization (Embo), plus simultaneous microsurgical resection (MSR) in the one-staged hybrid operating room. Patients who had received stereotactic radiosurgical treatments were excluded.

Ethical approvals were obtained from the IRBs of each contributing center. Written informed consent was acquired from all participants involved.

### Group Assignment

According to the surgical paradigm received, patients were divided into two groups: Embo+MSR and iDSA+MSR group. In the Embo+MSR group, patients received iDSA and endovascular embolization plus simultaneous microsurgical resection. In the iDSA+MSR group, patients received iDSA plus primary microsurgical resection.

### Preoperative Evaluation

Neurological evaluations were performed by neurosurgical physicians using modified Rankin's scale (mRS) at admission, discharge, and follow-up points. The magnetic resonance imaging (MRI) and DSA were performed in every patient for morphological and angiographic features of lesions. Functional MRI (fMRI) was additionally performed in patients with eloquent areas potentially involved. The lesion to eloquent distance (LED) was qualitatively measured in the neuro-navigation working station (Brainlab® Cranial 3.0, Brainlab AG, Munich, Germany). Eloquence included eloquent cortices, such as areas of the motor, Broca, Wernick, and the visuosensory, and fiber tracts, such as pyramidal tracts, arcuate fasciculus, and optic radiations. A LED <5 mm was defined as the threshold of a lesion with eloquence involved (25). Neuro-images were evaluated by one experienced radiologist, one neurosurgeon, and one interventional radiologist, independently. Multidisciplinary discussions were held to make individualized operative plans.

### Indications for Endovascular Embolization

Endovascular embolization was preferred in patients with the following indications: 1) high flow nidus (by frame-by-frame analyses of DSA);(26) 2) deep originated feeding arteries, accessible by microcatheters; 3) reduction of nidus size necessary; 4) diffusive lesion without clear boundaries; or 5) the need for eloquence protection.

### Implementation of One-Staged Hybrid Operation

The operations were performed in customized one-staged hybrid operating rooms (ORs). Each one-staged hybrid OR essentially consisted of a radiolucent operating table (MAQUE Holding B.V. & Co. KG, Rastatt, Germany), a surgical microscope (Pentero®900, Carl Zeiss Surgical AG, Oberkochern, Germany), and a monoplane angiographic system. ARTIS Pheno, ARTIS Zeego systems (Siemens Healthineers, Erlangen, Germany), and Allura Xper FD20 system (Philips Healthcare, Best, Netherland) were practicable for cerebrovascular one-staged hybrid operations.

Under general endotracheal anesthesia, electrophysiological changes were monitored via neurophysiological monitoring (IONM, Nicolet Endeavor CR IOM, Natus Medical Incorporated, CA, USA), including sensory evoked potentials, motion evoked potentials, and electromyography. The patient was supinely or laterally placed in the operative position, with the head fixed by a radiolucent head frame (MAYFIELD Infinity XR2 Radiolucent System, Integra LifeScience, Inc., NJ, USA). Operations were performed in one position. A 6-French(Fr) sheath (AVANTI® Introducer, Cordis Corporation, Miami Lakes, FL, USA) was used to establish the transarterial approach. A 6-Fr guide catheter (Envoy®, Codman division of Johnson & Johnson Medical Ltd., Wokingham, Berkshire, UK) was catheterized into the petrous segment of the internal carotid artery (ICA) or the foraminal segment of the vertebral artery (VA). Subsequently, the flow-dependent microcatheter (Marathon 1.3-F, eV3/Covidien, Minnesota, USA, or Excelsior SL-10, Stryker Neurovascular, California, USA, or Headway-17, MicroVention, California, USA) was advanced over a microwire (Synchro-14, Stryker Neurovascular, California, USA or Traxcess-14, MicroVention, California, USA) into the target feeding arteries under road-mapping. A confirming angiogram via the microcatheter was required before the liquid embolic agent (Onyx 18/34, eV3 Covidien, Minnesota, USA) was infused. When embolization was completed, the guide catheter was withdrawn from ICA and VA back to the ipsilateral common carotid artery (CCA) or subclavian artery, respectively. The Guide catheter was preserved with prolonged infusion of heparin saline (1000 IU/L, 0.6~1 ml/min) during the microsurgical operation. This could simplify the process of intraoperative DSA, especially when the patient was in a lateral position. Afterward, the microsurgical resection was performed as routine under the assistance of neuro-navigation (Brainlab® Cranial 3.0, Brainlab AG, Munich, Germany). Complete resections were achieved in all cases. While in some AVMs with eloquent areas involved, procedures of *in-situ* embolization combined with surgical resection were performed (27). Before closure, intraoperative DSA was performed via the preserved guiding catheter to ensure complete obliteration. Microsurgical resection would be repeated until there was no residual nidus observed.

### Follow-Up

Patients received the follow-up 3, 6, and 12 months after the operation for outcome evaluation. Most of the patients visited the outpatient department of Beijing Tiantan Hospital on the scheduled date and received physical and radiological examinations, such as CTA and DSA. The rest of the patients received interviews by telephone. All medical records and angiographic neuro-images were collected.

### Outcomes Evaluations

Deterioration of neurological deficits (DNDs) was the primary outcome, defined as a mRS increased during hospitalization and > 2 when discharged, including fatality (mRS = 6). Secondary outcomes included: 1) fatality, defined as the death related to operation or AVMs; 2) residue nidus, defined as the arteriovenous shunting found by postoperative angiograms in the operative field or neighborhoods; 3) intracranial hemorrhagic complications, defined as any emerging of high-density on postoperative cranial CT scan with a volume>5 ml (measured with ABC/2 method) within 7 days after the operation; 4) ischemic complications, defined as any low-density territory, except the operative region, revealed on CT scans within 7 days after the operation; 5) postoperative seizure, repeat or new onset of grand mal epilepsy; and 6) other complications, including infections in the central neural and respiratory system, thrombosis in deep veins, etc.

### Data Collection

All of the information was prospectively collected by full-time clinical research coordinators. All data were quality-controlled monthly by clinical research associates of a third-party contract research organization.

### Statistical Analysis

All statistical analyses were performed using IBM® SPSS® Statistics (Version 22, IBM, NY, United States). The baseline information was qualitatively and quantitatively described. The baseline of different groups was compared via normal distribution tests and non-parametric tests. Pearson chi-square tests and Fisher exact tests were performed to compare the differences of outcomes between groups.

## Results

Thirty-eight cases (male: female=23:15) with AVMs in SMG IV and V met the inclusion and exclusion criteria and were involved in our study (Baseline information refers to Table 1). Patients were in a mean age of 27.5 ± 15.98 y (ranged 5–63 y). The most common initial presentation was AVM rupture, which occurred in 26 cases (68.4%). The incidence of rupture history was higher in the Embo+MRS group than the iDSA+MRS group without any significant difference (75 vs. 57.1%, χ^2^ test, *P* = 0.217). Other initial presentations included neurological deficits in nine cases (23.7%), seizure in eight cases (21.1%), and incident in four cases (10.5%). Seven cases had received AVM-related treatment before being recruited, including the hematoma evacuation in six cases and endovascular embolization in one case. Thirty-two patients had a mRS score ≤ 2, including 21(87.5%) in the Embo+MSR groups and 11(78.6%) in the iDSA+MSR group, without significant difference (Mann–Whitney *U*-test, *P* = 0.178).

**Table 1:** Characteristics in patients with SMG IV and V bAVMs^*^.

**Variable**	**Overall**	**Intergroup**		
		**Embo+MSR**	**MSR+iDSA**	***P*** **-value**
Presentation				
No. of patients	38	24	14	
Male	23 (60.5)	15 (62.5)	8 (57.1)	0.505
Age in yrs	27.5 ± 15.98	28.6 ± 16.70	24.3 ± 15.19	0.559
Prior treatments				
Embo	1 (2.6)	1 (4.2)	0 (0)	0.615
Hematoma evacuation	6 (15.8)	4 (16.7)	2 (14.3)	0.615
Initial presentation				
Rupture	26 (68.4)	18 (75)	8 (57.1)	0.217
Neurological deficits	9 (23.7)	7 (29.2)	2 (14.3)	0.528
Seizure	8 (21.1)	3 (12.5)	5 (35.7)	0.102
Incidental	4 (10.5)	3 (12.5)	1 (7.1)	0.528
Headache	3 (7.9)	2 (8.3)	1 (7.1)	0.698
Preop. mRS score				0.178
0	4 (10.5)	4 (16.7)	0 (0)	
1	20 (52.6)	13 (54.2)	7 (50.0)	
2	8 (21.1)	4 (16.7)	4 (28.6)	
3	1 (2.6)	0 (0)	1 (7.1)	
4	4 (10.5)	2 (8.3)	2 (14.3)	
5	1 (2.6)	1 (4.2)	0 (0)	
Neuro-image				
Location				
Deep location	3 (7.9)	2 (8.3)	1 (7.1)	0.698
Multiple lobes	16 (42.1)	11 (45.8)	5 (35.7)	0.396
Diffuse	10 (26.3)	5 (20.8)	5 (35.7)	0.264
Feeding arterial circulation				
Anterior alone	16 (42.1)	8 (33.3)	8 (57.1)	0.201
Posterior alone	4 (10.5)	3 (12.5)	1 (7.1)	0.528
Both	18 (47.4)	13 (54.2)	5 (35.7)	0.304
Perforating artery	6 (15.8)	5 (20.8)	1 (7.1)	0.264
Draining vein				
Deep	12 (31.6)	8 (33.3)	4 (28.6)	0.528
Superficial	7 (18.4)	5 (20.8)	2 (14.3)	0.483
Both	19 (50.0)	11 (45.8)	8 (57.1)	0.369
Maximum diameter in centimeters	4.9 ± 1.36	5.1 ± 1.44	4.6 ± 1.19	0.297
Eloquence	35 (92.1)	21 (87.5)	14 (100)	0.240
LED in millimeters	2.6 ± 2.34	2.9 ± 2.64	2.0 ± 1.62	0.245
SMG				0.416
IV	33 (86.8)	20 (83.3)	13 (92.9)	
S2E1V1	24 (63.2)	13 (65.0)	11 (84.6)	
S3E1V0	6 (15.8)	4 (20.0)	2 (15.4)	
S3E0V1	3 (7.9)	3 (15.0)	0 (0)	
V	5 (13.2)	4 (16.7)	1 (7.1)	
Post-embolization SMG				
I	NA	1 (4.2)	NA	NA
S1E0V0		1 (4.2)		
II	NA	5 (20.8)	NA	NA
S1E0V1		2 (8.3)		
S1E1V0		1 (4.2)		
S2E0V0		2 (8.3)		
III	NA	9 (37.5)	NA	NA
S2E0V1		8 (33.3)		
S2E1V0		1 (4.2)		
IV	NA	7 (29.2)	NA	NA
S2E1V1		6 (25)		
S3E1V0		0 (0)		
S3E0V1		1 (4.2)		
V	NA	2 (8.3)	NA	NA
Outcome				
Postoperative complications				
Hemorrhage	3 (7.9)	3 (12.5)	0 (0)	0.240
Ischemia	2 (5.3)	2 (8.3)	0 (0)	0.393
CNS infection	6 (15.8)	3 (12.5)	3 (21.4)	0.385
Others	3 (7.9)	1 (4.2)	2 (14.3)	0.302
Second operation	2 (5.3)	2 (8.3)	0 (0)	0.393
Deterioration of neurological deficits (number of reaching the time point)				
Discharge (*n* = 38)	9 (23.7)	8 (33.3)	1 (7.1)	0.071
3-month FU (*n* = 37)	5 (13.5)	5 (20.8)	0 (0)	0.098
6-month FU (*n* = 37)	5 (13.5)	5 (20.8)	0 (0)	0.098
12-month FU (*n* = 37)	3 (8.1)	3 (12.5)	0 (0)	0.260

*Embo, Endovascular Embolization; MSR, Microsurgical resection; iDSA, Intraoperative Digital Subtraction Angiography; Prepo., Preoperation; mRS, Modified Rankin Scale; LED, Lesion to Eloquence Distance; SMG, Spetzler-Martin Grade; CNS, Central Nervous System; FU, Follow-up; NA, not applicable*.

* *Values are number of patients (%) unless noted otherwise*.

The localization and morphological information can be found in Table 1. Deep locations, such as basal ganglia and insula, were involved in three cases (7.9%). Diffusive nidus was observed in 26.3% of cases (*n* = 10). The maximum diameter of nidus was 4.9 ± 1.36 cm on average (ranged 3.0–7.5 cm). Thirty cases received the fMRI scan, while eight cases solely received the structural MRI scan. Eloquent areas were involved in 35 cases (92.1%) with an LED < 5 mm. In the aspect of angioarchitecture, arterial feeders that originated from solely anterior or posterior circulation were found in 17 (44.7%) cases and four cases (10.5%), respectively, and from both circulations in 17 cases (44.7%). The supply from perforating arteries was observed in six cases (15.8%), including anterior choroidal artery + lenticulostriate arteries in two cases, the anterior choroidal artery in three cases, and lenticulostriate arteries in one case. Nidus were drained from the deep draining vein(s) alone in 12 cases, superficial vein(s) alone in seven cases, and both in 19 cases. There were five cases (13.2%) with AVMs in SMG V, and 33 cases (86.8%) with AVMs in SMG IV, including 24 cases of S2E1V1, six cases of S3E1V0, and three cases of S3E0V1. The involvement of multiple lobes, the feeders from both anterior and posterior circulation, deep draining vein, larger maximum diameter, and the eloquent area was observed more frequently in Embo+MSR groups, but without significant difference (refer to Table 1).

All patients received selective operations. Fourteen cases (36.8%) underwent the paradigm of iDSA plus microsurgical resection. Twenty-four cases (63.2%) underwent the paradigm of endovascular embolization plus simultaneous microsurgical resection (details in Table 2, Case 4 and 11 refer to the section of Illustrative Cases). SMG grades were degraded via embolization in 15 cases (Table 2, Column 9 vs. Column 14). The degradations in nidus size and eloquent involvement were achieved in 11 cases, respectively. Residual nidus was detected via intraoperative DSA in two cases, which received immediate resections. The total operating time was 8.3 ± 2.06 h on average (ranged 5.52–15.17 h), in which 5.9 ± 2.08 h was for surgical procedures. The intraoperative bleed loss ranged from 150 to 6,000 ml (mean 1,153 ± 1282.8 ml, median 750 ml), including 150 to 4,800 ml (mean 1,000 ± 1263.5 ml) in iDSA+MSR group and 200–6,000 ml (mean 1,243 ± 1312.3 ml) in Embo+MSR group, without significant difference (student-*t*-test, *P* = 0.576). Intraoperative blood salvage was performed in 61.5% of cases (*n* = 24). Four cases received homologous blood products transfusion, including suspension of red blood cells in two cases, suspension of red blood cells+serum in one case, and fresh frozen plasma in one case. Doppler ultrasound was applied to 33 cases for detecting residues and reported two (6%) false-negative results.

**Table 2:** Summary of patients who underwent the paradigm of embolization plus simultaneous microsurgical resection.

**Case no**.	**Age** **(yr)**	**Gender**	**Surgical history**	**Initial symptom**	**Location**	**Feeding artery**	**Draining vein**	**SMG**	**Embolized feeders**	**Embolic agent (ml)**	**Balloon**	**Embo rate (%)**	**Post-** **embo SMG**	**Resection time (hr)**	**Blood loss (ml)**	**Compli-** **cation**	**Second Op**.	**Postoperative mRS score**					
																		**Adm**.	**Dis**.	**3 mo**.	**6 mo**.	**12 mo**.	**Other**
1	32	F	No	Incident	T P O	R.MCA +R.PCA	SDV +DDV	V	R.MCA	2.5	Unused	<50	S3E1V1	2.87	300	No	No	1	0	0	0	0	
2	16	M	No	Seizure	O	L.ACA +L.PCA	SDV	V	L.ACA	18	Unused	50–90	S3E1V1	6.38	1,900	No	No	0	0	0	0	0	
3	13	M	No	ICH	T	R.MCA +R.AChoA +R.LentA	SDV +DDV	S2E1V1	R.MCA	0.5	Unused	<50	S2E1V1	2.53	1,000	No	No	0	0	0	0	0	
4	12	F	No	ICH+ Dyskinesia	P	R.MCA +R.ACA	SDV +DDV	S2E1V1	R.ACA	1.2	Unused	<50	S2E1V1	5.08	750	No	No	1	3	2	1	1	
5	22	F	No	Seizure	Bg	R.MCA +R.PCA +R.AChoA	DDV	S2E1V1	R.PCA	0.5	Unused	<50	S2E1V1	2.65	300	Cerebral Infarction	No	1	4	4	3	3	
6	26	M	No	Seizure	T	R.MCA +R.PCA	SDV +DDV	S2E1V1	R.PCA	1.5	Unused	<50	S2E1V1	3.55	1,000	No	No	1	1	1	1	1	
7	39	M	No	ICH+ IVH	P O	L.ACA +L.PCA	DDV	S2E1V1	R.PCA	2.5	Unused	<50	S2E1V1	2.27	1,000	No	No	5	1	0	0	0	
8	45	F	No	Incident	F	L.ACA	SDV +DDV	S3E0V1	R.ACA	1	Unused	>90%	S3E0V1	6.57	2,000	No	No	1	4	1	1	1	
9	16	F	No	ICH	T P	R.MCA +R.PCA +R.AChoA	SDV +DDV	S2E1V1	R.MCA	1.1	Pressure cooker	50–90%	S2E1V1	2.00	400	No	No	1	1	0	0	0	
10	42	M	No	Headache	F P	R.ECA +R.MCA +R.PCA	DDV	S3E0V1	R.MCA +R.PCA+ R.MMenA	11.5	Unused	<50%	S2E0V1	5.82	6,000	CNS infection	No	1	0	0	0	0	
11	25	M	No	ICH	T P O	R.MCA +R.ACA +R.PCA	DDV	V	R.PCA	10.5	Unused	>90%	S2E0V1	5.45	1,600	Seizure	No	2	4	3	2	1	
12	45	F	No	ICH	P O	L.MCA +L.ACA +L.PCA	SDV	S3E1V0	L.ACA	12	Unused	>90%	S2E1V0	3.27	200	Hemorrhage	No	0	4	2	2	2	
13	32	M	HE +BHD	ICH+ IVH+ SAH	P O	RMCA +RACA +RPCA	SDV +DDV	S2E1V1	L.ACA	1.8	Unused	50–90%	S2E0V1	6.12	1,000	No	No	4	2	2	2	2	
14	6	M	HE	IVH+ SAH	P	RMCA +RAChoA +RLentA	SDV +DDV	S2E1V1	R.MCA +R.LentA	3	Pressure cooker	50–90%	S2E0V1	3.10	4000	No	No	1	2	1	0	0	
15	32	M	No	ICH	F	RMCA	SDV +DDV	S2E1V1	R.LentA	1.2	Temporal occlusion	<50%	S2E0V1	4.15	300	No	No	1	4	3	3	2	
16	10	M	No	ICH+ SAH	P	RMCA +RACA +RPCA +RAChoA +RPChoA +RLentA	SDV +DDV	V	R.MCA +R. LentA	9	Unused	>90%	S2E0V1	4.12	500	No	No	1	0	0	0	0	
17	14	M	No	IVH	F P	RACA	DDV	S2E1V1	R.ACA	1	Pressure cooker	50–90%	S2E0V1	3.68	500	No	No	2	2	1	1	1	
18	34	F	EE	ICH	C	LAICA +LSCA +LPICA	SDV	S3E1V0	L.SCA +L.PICA	4.5	Unused	>90%	S1E1V0	3.43	800	CNS infection	No	1	2	2	2	1	
19	53	M	No	ICH	T P	LPCA	SDV	S3E1V0	L.MCA +L.PCA	10	Unused	>90%	S2E0V0	6.20	2000	Cerebral hemorrhage +Cerebral infarction +Respiratory infection	7^th^ day	0	5	5	5	5	Dead in the 24^th^ mo.
20	48	M	HE	IVH+ Hypopsia	O	LACA +LPCA	SDV +DDV	S2E1V1	L.ACA +L.MCA +L.PCA	9	Unused	>90%	S1E0V1	5.55	500	No	No	2	2	2	2	2	
21	49	F	No	ICH	P O	RMCA	SDV	S3E1V0	R.MMenA	4.7	Unused	>90%	S2E0V0	3.73	1,500	Cerebral Hemorrhage	No	1	5	6	NA	NA	Dead in the 2^nd^ mo.
22	5	F	No	ICH	P O	LMCA +LPCA	DDV	S2E1V1	L.PCA	5	Unused	>90%	S1E0V1	2.22	500	No	No	1	0	0	0	0	
23	8	M	HE	ICH+ IVH+ Dyskinesia	Bg	LMCA +LPCA	DDV	S2E1V1	L.MCA	1.2	Unused	<50%	S2E0V1	5.15	800	CNS infection	No	4	4	4	3	3	
24	63	M	No	IVH	F	LACA	DDV	S3E0V1	L.ACA	2.5	Pressure cooker	>90%	S1E0V0	2.45	1,000	No	No	2	1	1	1	1	

*SMG, Spetzler-Martin Grade; Embo, Embolization; Op., Operation; mRS, Modified Rankin Scale; Adm., Admission; Dis., Discharge; mo., Months; HE, Hematoma Evacuation; BHD, Bur Hole Drainage; EE., Endovascular Embolization; ICH, Intracerebral Hemorrhage; IVH, Intraventricular Hemorrhage; SAH, Subarachnoid Hemorrhage; F, Frontal lobe; T, Temporal lobe; P, Parietal lobe; O, Occipital lobe; Bg, Basal Ganglion; C, Cerebellum; R., Right; L., Left; MCA, Middle Cerebral Artery; PCA, Posterior Cerebral Artery; ACA, Anterior Cerebral Artery; AChoA, Anterior Choroidal Artery; ECA, External Carotid Artery; LentA, Lenticulostriate Artery; PChoA, Posterior Choroidal Artery; AICA, Anterior Inferior Cerebellar Artery; SCA, Superior Cerebellar Artery; PICA, Posterior Inferior Cerebellar Artery; SDV, Superficial Draining Vein; DDV, Deep Draining Vein; MMenA, Middle Meningeal Artery; CNS, Central Nervous System; NA, Not Applicable*.

Intracranial hemorrhagic and ischemic complications occurred in three cases (7.9%) and two cases (5.3%), respectively. The second operation was performed in two cases (5.3%) to evacuate the postoperative hematoma. Infection of the central neural system occurred in six cases (15.8%). Infection of the respiratory system occurred in one case (2.6%), and postoperative seizure occurred in one case (2.6%).

Deterioration of neurological deficits occurred in 23.7% of cases (*n* = 9) when discharged. Among the patients who reached follow-up points of 3, 6, and 12 months, incidences of DNDs were 13.5% (5 out of 37), 13.5% (5 out of 37), and 8.1% (3 out of 37), respectively. Two deaths (5.3%) occurred in total, including one 2 months after the operation, and the other after 24 months in a coma. DNDs occurred frequently in Embo+MSR groups at every time point without significant differences (refer to Table 1).

Technical details, such as embolization rate and SMG degradation, were further investigated in subgroup comparisons (refer to Table 3). The operative difficulty was represented by blood loss and the microsurgical time. The clinical outcome was represented by the incidence of DNDs. Similar blood loss occurred in the patients receiving no embolization (1,000 ± 1263.5 ml) and near-total embolization (1,060 ± 667.0 ml, t-test, *P* = 0.893), but without significant difference compared to other patients who received partial (1,272 ± 1799.5 ml, t-test, *P* = 0.673) and subtotal embolization (1,560 ± 1487.6 ml, t-test, *P* = 0.427). Patients receiving near-total embolization had the shortest resection time but without a significant difference to the longest one (5.59 ± 1.471 h vs. 6.81 ± 2.441, t-test, *P* = 0.298). Patients receiving subtotal embolization had no deterioration of neurological deficits occur. By contrast, near-total embolization induced DNDs in 50% (*n* = 5) of patients at discharge (Fisher exact test, *P* = 0.028) and 20.0% of patients at 12-month follow-up (Fisher exact test, *P* = 0.178).

**Table 3:** Subgroup comparisons in different embolization rates and SMG degrading strategies.

**Category**	**Blood loss (ml)**		**Resection time (hrs)**		**Deterioration of neurological deficits (n/N)**						
	**Volume**	***P-*** **value**	**Duration**	***P-*** **value**	**Dis**.	***P-*** **value**	**3 mo.FU**	***P-*** **value**	**6 mo.FU**	**12 mo.FU**	***P-*** **value**
Embolization rate^*^											
Non-embolization	1,000 ± 1263.5		5.8 ± 2.40		1/14		0/13		0/13	0/13	
Partial (0–50%)	1,272 ± 1799.5	0.673	5.8 ± 2.14	0.982	3/9	0.147	2/9	0.156	2/9	1/9	0.409
Subtotal (51–89%)	1,560 ± 1487.6	0.427	6.8 ± 2.44	0.419	0/5	0.737	0/5	NA	0/5	0/5	NA
Near-total (≥90%)	1,060 ± 667.0	0.893	5.6 ± 1.47	0.836	5/10	0.028	3/10	0.068	3/10	2/10	0.178
**SMG degradation**											
Size						0.217		0.150			0.289
−2 points	900 ± 141.4^†^		4.7 ± 0.74^†^		0/2		0/2		0/2	0/2	
−1 point	1,511 ± 1792.0	0.655	6.1 ± 1.96	0.348	4/9		3/9		3/9	2/7	
−0 point	1,053 ± 1137.3	0.852	5.9 ± 2.20	0.471	5/27		2/26		2/26	1/26	
Eloquence (*n* = 35)						0.194		0.029			0.239
−0 point					4/24		1/23		1/23	1/23	
−1 point					4/11		4/11		4/11	2/11	

*n/N, Incident number /Total number at the time point; Dis., Discharge; mo., Month; FU, Follow-up; iDSA, Intraoperative Digital Subtraction Angiography; SMG, Spetzler-Martin Grade*.

* *Each of subgroups was compared with non-embolization subgroup (iDSA alone)*.

† *The baseline of intergroup comparisons*.

The embolization degraded the scores of SMG on size and eloquence (details refer to Table 3). The patients who decreased by 2 points on size obtained the most optimal outcome, with 900 ± 141.4 ml of blood loss, 4.70 ± 0.742 h of resection, and no incidence of DNDs. The embolization of eloquent area, −1 point in Eloquence, induced more occurrence of DNDs, especially in 3- and 6-month follow-up (−0 point vs. −1 point = 1/23 vs. 4/11, Fisher exact test, *P* = 0.029).

## Discussion

In this study, one-staged hybrid operations were performed to treat high-grade brain arteriovenous malformations on 38 patients. The applicable paradigm consisted of intraoperative digital subtraction angiography, endovascular embolization, and microsurgical resection. Two cases of residue (5.3%) were detected by iDSA, which were false-negative in intraoperative Doppler sonography. Complete obliterations were achieved in all cases. Deterioration of neurological deficits occurred in 23.7% of cases at discharge, and 13.5, 13.5, and 8.1% of patients at 3-, 6-, and 12-month follow-ups, respectively. The postoperative hemorrhage occurred in 7.9% of cases. The therapeutic paradigms based on one-staged hybrid operation were revealed to be feasible in treating high-grade AVMs.

The high-grade AVM is a critical challenge for the cerebrovascular surgeon. Difficulties lie in achieving both complete obliteration and neurological protection at the same time. Different therapeutic modalities have been proposed, including the initial microsurgical resection, and later staged multimodality treatments. Microsurgical resection is the early method of treating AVMs and was reported to achieve a complete obliteration rate of 96% (28). Still, it was not recommended to be primarily performed on high-grade AVMs, for its incidence of DNDs ranging from 31 to 38% (29, 30). Different types of multimodality treatments were reported to be used to treat most of the high-grade AVMs in current studies, including 1) endovascular embolization plus stereotactic radiosurgery (EE+SRS), 2)stereotactic radiosurgery plus delayed microsurgical resection (SRS+dMSR), and 3) staged endovascular embolization plus microsurgical resection (sEE+MSR). Blackburn et al. (31) reported their experience of performing the paradigm of EE+SRS without any incidence of neurological deficits. However, it should be noticed that the obliteration rate of the EE+SRS paradigm was reported to be 81%, which might expose patients to hemorrhagic risks due to residual nidus (5, 7). SRS+dMSR is another paradigm for treating high-grade AVMs. In this paradigm, the preoperative SRSs gradually eliminate parts of nidus and feeders over 2–3 years, and the delayed microsurgery ensured the complete elimination of the lesion. Abla et al. (20) reported their experience on using the SRS+dMSR paradigm in 16 cases and achieved an obliteration rate of 93.8% and a DNDs incidence of 50%. Though the paradigm was proved to be feasible, the hemorrhagic risk in latency periods of SRS still should be noticed, which could reach an incidence as high as 10–25.7% (15–19, 32). The paradigm of sEE+MRS is the most commonly used modality on treating high-grade AVMs. The preoperative endovascular embolization could decrease the blood volume of the lesion to reduce the operative risk in the following microsurgery (33). The MRS guaranteed a high complete obliteration rate but resulted in a DNDs incidence ranging from 24 to 63% (21–23). The staged treatment would expose the patient to a risk of intracranial hemorrhage after embolization ranging from 11 to 22.7% (34–36). In this study, 100% of complete obliteration rate and 8.1% of neurological deficit incidence in 12-month follow-up were achieved in one-staged paradigms. Though higher DND incidence occurred in EE+MRS groups compared to the iDSA+MRS group (0 vs. 12.5%, *P* = 0.260), both modalities resulted in acceptable clinical outcomes, without limitations in staged paradigms. Meanwhile, it should be noticed that 68.4% of patients presented with ruptured AVMs, which had been proven to be a protective factor of neurological outcomes (25). Intraoperative DSA played an important role in one-staged paradigms. It remedied the false-negative results of intraoperative Doppler sonography and ensured the complete obliteration of AVMs.

Endovascular embolization is believed to reduce operative risks and improve clinical outcomes of AVMs. EE+MRS group achieved relatively worse neurological outcomes and higher operative risks (more blood loss) than the iDSA+MRS group in every assessment point (refer to Table 1) in our study. Though there was no significant difference between groups on the baseline, AVMs in the EE+MRS group had higher proportions in the involvement of multiple lobes (45.8 vs. 35.7%), feeders of both anterior and posterior circulation (50 vs. 35.7%), deep draining vein (33.3 vs. 28.6%), and larger nidus (5.1 ± 1.44 cm vs. 4.6 ± 1.19 cm). As the data extracted from a real-world registry, participants in the EE+MRS group ought to harbor the lesion with more complex morphological and angioarchitectural conditions, which lead to worse clinical outcomes than those in the iDSA+MRS group. Despite that, two groups in this study achieved results without significant difference.

### Technical Details of Paradigm

According to the practical experience of this study, the following technical details might influence the neurological outcomes of patients who use this paradigm.

#### Embolization Rate

In this paradigm, the procedure of embolization was aimed at reducing the risk and difficulty of subsequent microsurgical resection by occluding the targeted feeders and nidus, rather than achieving complete obliteration. The arterial feeders and nidus, difficult to control during microsurgery, were the prior targets of embolization. Due to varieties of hemodynamic status, different embolization rates were achieved when the targets above were occluded. Embolization rates were sorted into four categories: non-embolization (received DSA only), partial embolization (with a rate ≤ 50%), subtotal embolization (with a rate >51% and <90%), and near-total embolization (with a rate ≥90%). The neurological outcomes of classes were revealed to be different between categories. The minimum events of DNDs occurred in the subtotal embolization group (0 out of 5), and secondarily in the non-embolization group (1 out of 14). The near-total embolization group had the maximum DNDs events (5 out of 10) compared to non-embolization (50 vs. 7.1%, Fisher exact test, *P* = 0.028). The hemodynamic status of nidus changed with the increasing embolization rate, and further influenced the casting of the embolic agent. It resulted in the distinctions between different embolization rates, and further led to different neurological outcomes. In the partial embolization group, the majority of embolic agents coalesced in the nidus near its inflow entrance and rapidly reversed into the arterial feeder. It made little contribution to reducing microsurgical risks and difficulties, and made the least impact on neighboring parenchyma. In the subtotal embolization group, the microcatheter was primarily superselected into the nidus through the dominant feeding artery to infuse the embolic agent. Blood flow of the dominant artery could help the embolic agent widely cast out into the nidus, and avoid its premature retrograde casting into the superselected artery. The embolic agent was being intermittently infused into the nidus until target nidus or feeders were embolized, which was always achieved at an embolization rate > 50%. In the subtotal embolization group, the embolized structures were a portion of the nidus and the arterial segments approximal to the nidus. Near-total embolization shared the same process with subtotal embolization in its early phase, but would not stop until most of the nidus was radiologically obliterated with obvious retrograde embolization in most of the feeding arteries. It could help to decrease the bleeding in microsurgery but might injure neighboring arterial branches and parenchyma. According to our results, subtotal embolization was revealed to be the most effective on reducing the risk and difficulty of microsurgery and improving the neurological outcome. Nevertheless, partial and near-total embolization achieved success in some of the cases. Their application scenes remain to be further explored.

#### Embolization of Perforating Artery

Perforating arteries are proven to be a risk factor for microsurgical resection of AVMs (37, 38). These arteries are deep-originated to eloquent territories, which are difficult to expose and control. The targeted embolization could get the perforating arteries controlled ahead of surgical operation, which was applied to three cases (Case 14, 15, and 16). In Cases 14 and 16, perforators were embolized retrogradely from the nidus to the artery. In Case 15, the lateral lenticulostriate artery was embolized through a microcatheter, which had been super-selected from the embolic agent and was infused directly through the microcatheter, which was super-selected in it. The neurological function deterioration occurred in Case 15 but was not observed in the other two cases. Referring to technical details of three cases, the retrograde infusion of the embolic agent through nidus to perforators might be a safer manipulating method, which limited the embolic agent in the segment of perforator approximal to the nidus. By contrast, the embolization through a microcatheter super-selected into the perforator might impact neurological functions. Also, the manipulation of super-selection might be at risk of damaging the perforator and its branches.

#### AVM Degradation

High-grade AVM could be degraded by the intraoperative embolization in this paradigm. The degradation could be achieved by decreasing the point on size (by reducing the nidus size) and eloquence (increasing the distance between lesion and eloquence). The point on deep draining vein could hardly be changed since the premature occlusion of outflow would increase the rupture and edema risk of the lesion and the parenchyma around (39).

Degradations on size induced different neurological outcomes. Referring to Table 3, DNDs occurred in 0% of patients who decreased by 2 points in size, while 44.4% (4 out of 9) of patients decreased by 1 point. Only large AVMs (maximum diameter≥6 cm) could achieve a reduction of 2 points with an embolization rate of>50%. The neurological outcomes and operative difficulties of large AVMs might be improved by the sufficient reduction of their nidus. While, the decreasing of size by 1 point indicated a minor impact on the nidus, which made little contribution to operative risks and outcomes.

The degradation of eloquence is aimed at increasing lesion-to-eloquent distance (LED) to decrease the incidence of DNDs. Jiao et al. reported a cutoff LED of 5 mm, beyond which eloquent areas could be survived (25). However, in our study, the embolization near eloquence failed to improve the neurological outcome of patients when discharged. On the contrary, it resulted in worse outcomes over 6 months, compared to those with left eloquence score unchanged (discharge, −0 vs. −1=16.7 vs. 36.4%, Fisher exact test, *P* = 0.194; 6 months, −0 vs. −1= 4.3 vs. 36.4%, Fisher exact test, *P* = 0.029). The operative details revealed that the degradation of eloquent areas would not affect the neurological outcome alone. The strategy of subsequent microsurgery determined the success of neurological protection. Resecting the nidus along its initial boundary would still damage the parenchyma of eloquence nearby. In contrast, resecting in the embolized nidus would leave a sufficient LED between cutting edge and eloquence and create minimized neurological deficit. This technique, which preserves the embolized nidus near eloquence *in situ* and resected the rest, has been successfully applied to protect the neurological functions of patients (27, 40).

#### Illustrative Cases

##### Illustration of Case 4

**Figure 1:**
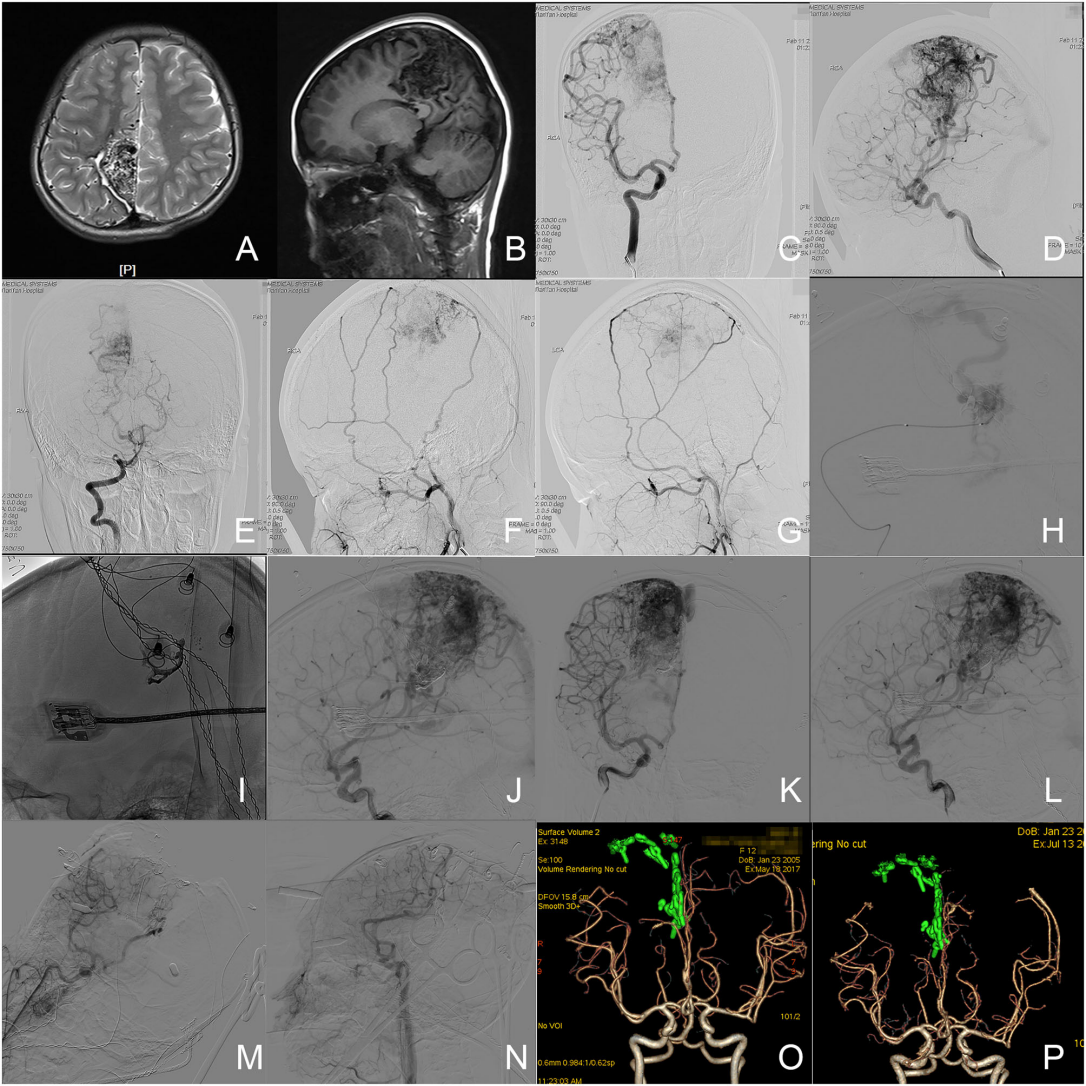
Illustration of case 4. **(A,B)** The axial T2-weighted and sagittal T1-weighted MR images revealed a cerebrovascular lesion in the right parietal lobe with isthmus of cingulate gyrus, parahippocampal gyrus, and splenium of corpus callosum involved. **(C,D)** Angiograms of right internal carotid artery (ICA) revealed the arterial feeders from the right arteries of central and postcentral sulcus, and the right pericallosal artery, paracentral artery, and precuneal artery, as well as drainage through Galen's vein and superficial draining vein. **(E)** Angiogram via the right vertical artery revealed the arterial feeders from the right posterior cerebral artery. **(F,G)** Angiograms via the bilateral external carotid arterial revealed the arterial feeders from the bilateral middle meningeal arteries. **(H)** An angiogram through the microcatheter (Excelsior SL-10, Stryker Neurovascular, California, USA) in the right pericallosal artery. **(I)** The casted embolization agent (high density object). **(J)** Angiogram through the right ICA in the work position after embolization. **(K,L)** The angiograms through the right ICA before microsurgery revealed the partial occlusion of nidus and feeder in the deep. **(M,N)** The intraoperative angiogram before closure confirmed the complete elimination of nidus. **(O)** The follow-up CTA 3 months after the operation revealed a complete elimination of nidus. **(P)** The follow-up CTA 17 months after the operation revealed no residue or recurrence. The green objects in **(O,P)** were the reconstructed clips.

A 12-year-old female presented with sudden weakness in her left extremities 10 years ago. Her head MRI revealed a bAVM located in the parietal lobe and involved the isthmus of the cingulate gyrus, parahippocampal gyrus, and splenium of the corpus callosum. Her dyskinesia was relieved after a partial endovascular embolization performed in a local hospital. The patient suffered a sudden headache accompanied by nausea and vomiting 2 months before admission. The patient complained about the episodic headache upon admission, and got a 1 score of mRS without any neurological deficit. A DSA was repeated for preoperative preparation. The nidus was mainly supplied by arterial feeders from the right arteries of the central and postcentral sulcus, and the right pericallosal artery, paracentral artery, and precuneal artery. The recruitment of arterial feeders from the right posterior artery and bilateral middle meningeal arteries could be observed. The nidus was drained via Galen's vein and superficial draining vein. In a one-staged hybrid operation, an embolization was firstly performed to eliminate the medial inferior nidus through the pericallosal artery to control the feeders from deep within the operative field. The microsurgical resection was performed after achieving a partial embolization. An intraoperative DSA was performed before closure and confirmed the complete elimination of nidus. The left extremities of the patient were paralyzed after the operation and improved to be capable of walking independently with a crutch (3 scores of mRS) when discharged after 20 days. The patient visited the outpatient department 3 months after the operation with slight weakness in the left extremities (2 scores of mRS). A 3-month follow-up CTA revealed no residue or recurrence of bAVM. The 6-and 12-month telephone follow-ups reported the recovery of strength in the left extremities and clumsiness of the left fingers (1 score of mRS). The patient visited the outpatient department again after 17 months with her left finger clumsiness unchanged. A 17-month follow-up CTA further confirmed the complete elimination of bAVM. Images of this case refer to Figure 1.

##### Illustration of Case 11

**Figure 2:**
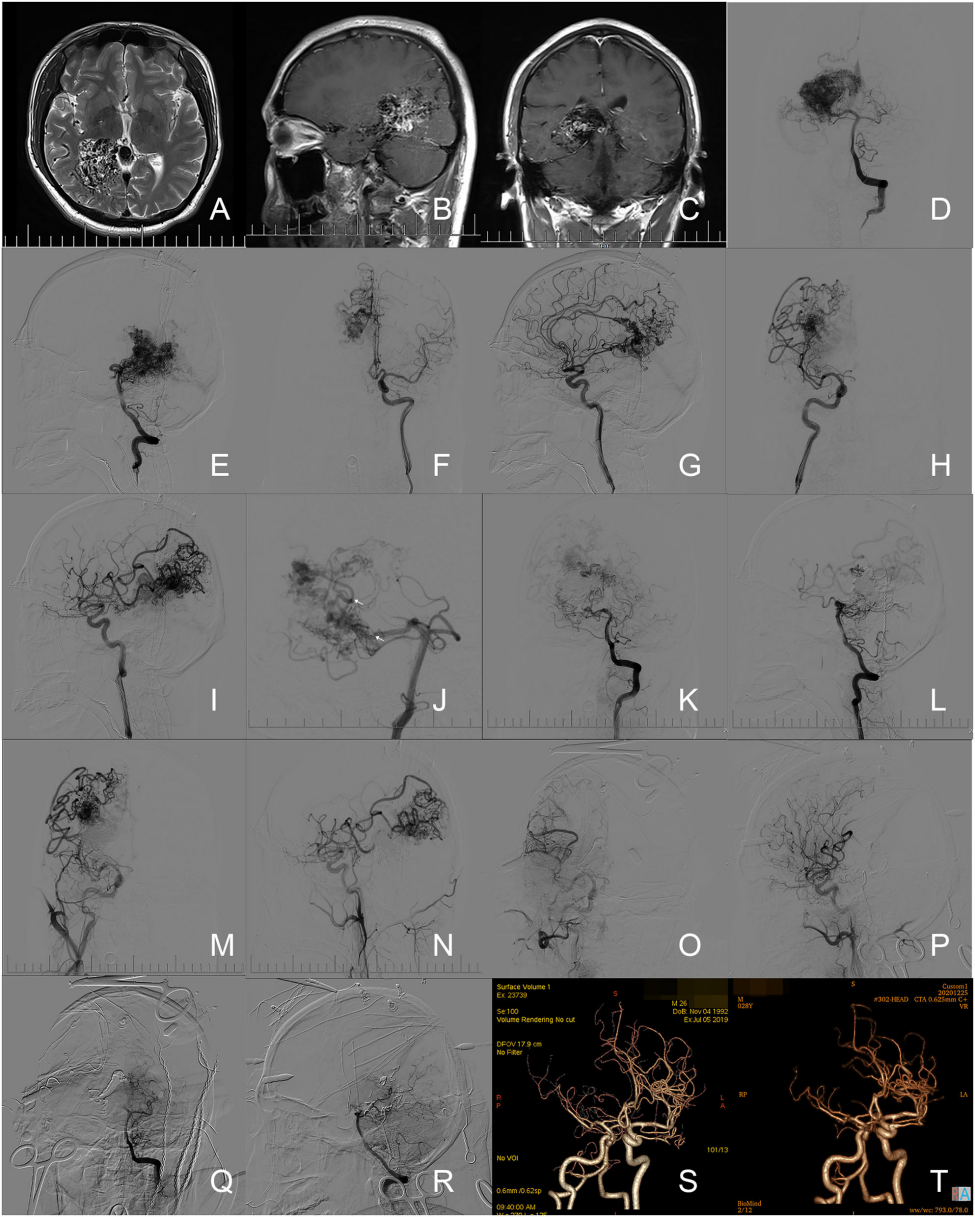
Illustration of case 11. **(A–C)** Preoperative axial T2-weighted image, sagittal, and coronary enhanced T1-weighted image of MR revealed the bAVM located in the right tempo-parietal-occipital lobe. **(D,E)** Angiograms via the left vertical artery revealed the feeders from the right posterior cerebral artery (PCA) and the drainage through Galen's vein. **(F,G)** Angiograms from the left ICA demonstrated the feeders from the right callosomarginal artery and precuneal artery. **(H,I)** Angiograms from the right ICA demonstrated the feeders from the right posterior parietal artery, postcentral sulcus artery, and anterior choroidal artery. **(J)** A microcatheter (Marathon 1.3-F, eV3/Covidien, Minnesota, USA. Arrows indicates the marker on the tip of the microcatheter) was super-selected into the nidus through the right PCA. **(K,L)** Angiograms through the left vertical artery suggested the occlusion of most arterial feeders from the right PCA. **(M,N)** Angiograms from the right ICA demonstrated the feeders from the anterior circulation. **(O-R)** The intraoperative angiograms through right ICA and left vertical artery confirmed the complete elimination of nidus. **(S)** A follow-up CTA at the 12th month after operation revealed no residue. **(T)** A follow-up CTA at the 30th month after operation revealed no recurrence of nidus.

A 25-year-old male complained about a sudden headache followed by a loss of consciousness 11 years ago. The patient was admitted into a local hospital and received a head CT and DSA. A rupture of bAVM in the right tempo-parietal-occipital lobe was discovered, but without any surgical intervention given. The patient remained dizzy, with blurred vision, and had stiff limbs when discharged from the local hospital. He visited our outpatient department for the deterioration of the symptoms above. The physical examination revealed blurred vision and clumsiness of distal extremities upon admission (2 scores of mRS). A head MRI revealed the lesion in the right tempo-parietal-occipital lobe. In the one-staged hybrid operation, the DSA revealed the nidus to be mainly fed by parietooccipital and calcarine branches of the right posterior cerebral artery (PCA). Other arterial feeders included the right callosomarginal artery, precuneal artery, posterior parietal artery, postcentral sulcus artery, and anterior choroidal artery. Nidus was drained by Galen's vein. Endovascular embolization was performed through the right PCA to eliminate the deep nidus and achieved about 50% embolization rate. Most of the feeders from the PCA were occluded with those from the anterior circulation remaining. The lesion was removed in the subsequent microsurgery. The complete elimination of bAVM was confirmed by the intraoperative DSA before closure. The patient was discharged 18 days after the operation with a critical weakness in the left limbs and deficit of vision field (4 scores of mRS). The telephone follow-up reported the sequential improvement in movement capability of left limbs and 3 and 2 scores of mRS after 3 and 6 months, respectively. The patient visited the outpatient department 12 months after the operation with only partly homonymous hemianopia remaining (1 score of mRS). There was no residue or recurrence of nidus found in the CTAs at 12 and 30 months. Images of this case refer to Figure 2.

### Modification and Expectation

The one-staged hybrid operation, which combines endovascular and microsurgical techniques, has been used in neurosurgery for over 5 years (41). It provides extraordinary surgical solutions to AVMs. It also makes it possible to account for the timing of subsequent microsurgery after embolization, the hemodynamic changes in operation, the predictors of normal perfusion pressure breakthrough and neurological deficit, etc. In this study, the practicability of one-staged hybrid operation on treating high-grade AVMs was validated. Meanwhile, technical details which might improve the operative safety and neurological outcomes were analyzed and proposed. These findings would be further studied in following specific studies with a larger sample size.

### Limitation

In this study, 38 cases with high-grade AVMs were enrolled. It met the requirement of describing the practicability of the paradigm based on the one-staged hybrid operation on treating high-grade AVMs, but could not make effective comparisons between different procedures and technical details. Further studies with a larger sample size are needed.

## Conclusions

The paradigms based on the one-staged hybrid operation were practical and effective in treating high-grade AVMs. Appropriate intraoperative embolization could help decrease operative risks and difficulties and improve neurological outcomes.

## Data Availability Statement

The raw data supporting the conclusions of this article will be made available by the authors, without undue reservation.

## Ethics Statement

The studies involving human participants were reviewed and approved by IRB of Beijing Tiantan Hospital, Capital Medical University. Written informed consent to participate in this study was provided by the participants' legal guardian/next of kin.

## Author Contributions

MW, FL, and HQ designed and conceptualized this work and participated in the data collection. MW participated in drafting the manuscript. YC, SW, and JZ critically revised the manuscript for important intellectual content. All authors contributed to the article and approved the submitted version.

## Funding

This work was funded by the Beijing Municipal Science and Technology Project (No. D161100003816005) and the National Key Technologies R&D Program of China (No. 2016YFC1301800). This study was supported by the China National Clinical Research Center for Neurological Diseases, Chinese Cerebrovascular Neurosurgery Society, and Chinese Interventional & Hybrid Operation Society of Chinese Stroke Association.

## Conflict of Interest

The authors declare that the research was conducted in the absence of any commercial or financial relationships that could be construed as a potential conflict of interest. The handling editor has declared a shared parent affiliation with the authors at the time of review.

## Publisher's Note

All claims expressed in this article are solely those of the authors and do not necessarily represent those of their affiliated organizations, or those of the publisher, the editors and the reviewers. Any product that may be evaluated in this article, or claim that may be made by its manufacturer, is not guaranteed or endorsed by the publisher.
